# Oxidized High-Density Lipoprotein and Cardiovascular Risk in Rheumatoid Arthritis

**DOI:** 10.14740/jocmr6494

**Published:** 2026-05-31

**Authors:** Linda Scharow, Susann Patschan, Nikolaos Pagonas, Meike Hoffmeister, Inga Claus, Emily Deichsler, Werner Dammermann, Oliver Ritter, Daniel Patschan

**Affiliations:** a Department of Cardiology und Rhythmology, University Clinic Ruppin-Brandenburg, Brandenburg Medical School (Theodor Fontane), Neuruppin, Brandenburg, Germany; b Department of Internal Medicine I - Cardiology, Nephrology and Internal Intensive Medicine, Brandenburg University Hospital, Brandenburg Medical School (Theodor Fontane), Brandenburg an der Havel, Brandenburg, Germany; c Institute of Biochemistry, Brandenburg Medical School (Theodor Fontane), Brandenburg an der Havel, Brandenburg, Germany; d Department of Medicine 2, Gastroenterology, Diabetes, Endocrinology, University Hospital Brandenburg of the Brandenburg Medical School Theodor Fontane, Brandenburg, Germany

**Keywords:** oxHDL, RA, Cardiovascular risk, DMARDs, Disease activity

## Abstract

**Background:**

Patients with rheumatoid arthritis (RA) have a disproportionately increased cardiovascular risk (CVR). Serological CVR predictors that capture this disease-associated risk increase are currently not available. Elevated levels of oxidized high-density lipoprotein (oxHDL) have been associated with coronary artery disease and atrial fibrillation. The aim of this study was to evaluate the suitability of oxHDL as a CVR predictor in RA.

**Methods:**

An observational cross-sectional study was conducted in patients with confirmed RA undergoing variable disease-modifying anti-rheumatic drug (DMARD) therapy. Anamnestic, clinical, and laboratory surrogate markers of cardiovascular morbidity and disease activity were collected. Th quantification of oxHDL was conducted using a fluorometric biochemical cell-free assay.

**Results:**

A total of 200 RA patients were included in the study. oxHDL showed significant correlations with the following variables: body mass index (BMI) (positive), total cholesterol, and average daily prednisolone dose (both negative). Overweight and hypertensive individuals exhibited higher levels of oxHDL. Additionally, oxHDL correlated positively with the number of tender small joints.

**Conclusion:**

oxHDL is associated with distinct CVR indicators in RA. Both prospective and follow-up data are needed to definitively evaluate the role of oxHDL in CVR assessment in RA.

## Introduction

Patients with rheumatoid arthritis (RA), the most prevalent inflammatory rheumatic disease, are not only at risk due to potentially disabling joint remodeling processes, but also due to a significant increase in the risk of cardiovascular disease and death. This connection can now be considered established, and official recommendations for cardiovascular diagnosis and treatment in RA have already been published [1]. Initially, RA patients accumulate conventional cardiovascular risk (CVR) factors similarly to individuals without systemic autoimmune disorders. However, the disproportionate increase in CVR is primarily attributed to the use of proatherogenic substances, such as glucocorticoids and non-steroidal anti-inflammatory drugs (NSAIDs), as well as the systemic effects of chronic inflammation associated with RA [2]. Consequently, new CVR scores, like QRISK3 [3], include the presence of RA as an independent risk factor. The QRISK3 also proved to be a potent tool for identifying patients with ankylosing spondylitis and increased CVR in recent work [4].

The analysis of lipid status is an essential part of CVR screening. An increase in the high-density lipoprotein fraction (HDL) is known to be associated with CVR reduction. While this association is well-established, the oxidized isoform of HDL (oxHDL) has been discussed to serve as a more precise predictor of cardiovascular events than the total quantity or concentration of HDL [5–7]. The oxidative modification of HDL is triggered by reactive oxygen species and lipid peroxidation radicals, leading to the transformation of HDL into dysfunctional, pro-inflammatory, and pro-atherogenic oxHDL [8, 9]. Unlike native HDL, which promotes reverse cholesterol transport and has anti-inflammatory and antioxidant effects, oxHDL loses these protective functions and becomes pro-inflammatory and pro-atherogenic [10]. Among patients with dyslipidemia, levels of oxHDL have been positively correlated with total HDL cholesterol (HDL-C) [11], suggesting that in certain high-risk contexts a greater total amount of HDL may provide more substrate for oxidative modification. In a 2023 study [12], oxHDL, in contrast to oxidized low-density lipoprotein (oxLDL), was significantly associated with the so-called fibrous burden, i.e. the atherosclerotic plaque burden, over a period of 3.3 years. Therefore, oxHDL may offer certain diagnostic advantages.

The modified form of HDL is subsequently ingested by macrophages, which differentiate into foam cells, a key component of early atherosclerotic plaques [13–15]. To date, increased oxHDL levels were shown in patients with ST-segment elevation myocardial infarction [16], atrial fibrillation (AF) [17], and aortic valve stenosis [18]. In an unpublished in-house study, this parameter has also been identified as a predictor of mortality in patients with newly diagnosed acute kidney injury. To our knowledge, only few investigations on oxHDL in patients with inflammatory rheumatic diseases have been published to date. One study included 33 individuals with RA, seven patients with axial spondyloarthropathy, and four individuals with systemic lupus erythematosus. A higher oxHDL was found in the overall collective examined than in the controls [19]. This study demonstrated that systemic concentrations of oxHDL are altered not only in RA but also in other systemic autoimmune diseases that are etiologically and phenotypically highly heterogeneous. A study published in 2023 also showed lower antioxidant capacity of HDL in RA [20].

The aim of the current study was to analyze oxHDL in only RA. The primary aim was to identify associations between the parameter and CVR surrogate markers. Secondarily, we aimed to investigate possible associations with indicators of disease activity.

## Materials and Methods

The studied cohort has already been described in a recently published study [21]. Therefore, the clinical characteristics are consistent.

### Design

This observational, cross-sectional, single-center study was conducted at the Health Center of Brandenburg University Hospital (Brandenburg Medical School Theodor Fontane) between November 2022 and January 2023. Formal approval was obtained from the Ethics Committee of the Brandenburg Medical School (Neuruppin, Brandenburg, Germany; Approval Number: E-01-20200316). The study adhered to the principles outlined in the Declaration of Helsinki (1975, as revised in 2013). To protect participant confidentiality, all medical and patient data were de-identified, ensuring that individuals could not be identified. The reporting of this study adheres to the STROBE guidelines [22].

### Patients

The patients who constituted the subject population of the study were initially identified through screening and subsequently recruited from the Health Center of the Brandenburg University Hospital, which is one of three university hospitals that are affiliated with the Brandenburg Medical School in Germany. Patients who met the following criteria were deemed eligible for inclusion in the study: 1) patients must have been 18 years of age or older at the time of enrollment, and 2) patients must have met the ACR/EULAR 2010 rheumatoid arthritis classification criteria [23]. Furthermore, the therapeutic regimen for the patients included a disease-modifying anti-rheumatic drug (DMARD) therapy, which necessitated the incorporation of either no DMARD (DMARD-native) or a minimum of one conventional or biologic DMARD. The daily dosage of prednisolone was subject to adjustment in accordance with the severity of disease activity. The following criteria were employed for the exclusion of patients from the study: the presence of uncontrolled psychiatric disorders, the existence of additional autoimmune-mediated diseases, the presence of uncontrolled infectious diseases such as human immunodeficiency virus (HIV), hepatitis B or C, and tuberculosis, the presence of uncontrolled drug or alcohol dependence, and the presence of pregnancy.

The patient characteristics that were collected included height, weight, comorbidities, medications, smoking status, and family history of cardiovascular disease. Disease activity was assessed using the Disease Activity Score in 28 joints-C-reactive protein (DAS28-CRP) score, with remission, low, moderate, and high disease activity defined by scores of < 2.6, 2.6 to 3.2, 3.2 to 5.1, and > 5.1, respectively. To assess disease activity, additional tools were employed, including the Visual Analogue Scale (VAS), which ranges from 0 (no pain) to 10 (maximum pain imaginable), a joint assessment for swelling and pain, and the Hannover Functional Questionnaire (HFQ) [24]. The data collected encompassed various aspects related to therapy, including the current DMARD therapy (agent), the use of NSAIDs (dosage and frequency of use), and the daily prednisolone dose in milligrams. Possible correlations or associations with the following indicators of RA disease activity were finally analyzed: VAS, HFQ, DAS28, CRP, and platelet count. Additionally, comparisons were made between individuals who tested positive and negative for rheumatoid factor (RF) and anti-citrullinated protein antibody (ACPA).

The CVR assessment included the following morbidities and laboratory parameters: arterial hypertension, diabetes mellitus including HbA1c (%), past and current smoking (at least 10 cigarettes per day), alcohol consumption (no alcohol, 1–3x per month, 1–3x per week, and daily). Physical activity was categorized as “no physical activity” and “physical activity,” with the following definitions: none, 2–3 times a week, and daily. Total cholesterol (mmol/L), LDL (mmol/L), HDL (mmol/L), and lipoprotein(a) (Lp(a), nmol/L) were also measured. The laboratory parameters measured included RF and ACPA titer, CRP levels (mg/L), complete blood count, and serum creatinine (µmol/L). The following metrics and nominal variables were finally defined and analyzed as CVR factors and morbidities: age, gender, body mass index (BMI), average daily prednisone dosage, serum creatinine, HbA1c, total cholesterol, LDL cholesterol, smoking status, physical exercise, distress, hypertension, known coronary artery disease, known heart failure, family history of atherosclerosis, diabetes mellitus, and obesity.

### Quantification of oxHDL

The quantification of oxHDL in serum was conducted using a validated fluorometric biochemical cell-free assay. This assay measures HDL lipid peroxide content based on the oxidation of the fluorochrome Amplex Red. Initially, the serum was depleted of apoB through the process of polyethylene glycol (PEG) precipitation. Subsequently, 50 µL of apoB-depleted serum was added to wells of a 96-well plate in duplicate, followed by the addition of 0.075 units per well of horseradish peroxidase (HRP) and 50 µM Amplex Red reagent, yielding a total volume of 100 µL. HRP has been demonstrated to catalyze the reaction of Amplex Red to resorufin in combination with endogenous peroxides. Following a 1-h incubation period, the fluorescence of resorufin at wavelengths of 535/590 nm was measured using a Spark 10M microplate reader (Tecan). In order to standardize the assay and minimize experimental variability, apoB-depleted sera from 10 healthy volunteers (who were not study participants) were pooled and utilized as experimental control in each plate. The mean fluorescence from each sample was then normalized by the mean fluorescence readout of the pooled control and HDL-C using the following calculation: nHDLox = (HDLox sample × 47 (mg/dL))/(HDLox control × HDL-C sample (mg/dL)), where 47 mg/dL represents the HDL-C of the pooled serum control. The samples were subsequently analyzed following the recruitment of the final patient. The intra-assay coefficient of variation (CV) was 6.7%. The inter-assay coefficient of variation was 3.7%. All laboratory measurements were conducted in the laboratory of the Department of Cardiology und Rhythmology, University Clinic Ruppin-Brandenburg, Brandenburg Medical School (Theodor Fontane), Neuruppin, Brandenburg.

### Statistical analysis

Comparisons between nominal data were conducted using the Chi-square test. Metric data were first assessed for normal distribution using the Kolmogorov-Smirnov test. For two-group comparisons, the Mann-Whitney U test was employed, while comparisons among more than two groups were performed using the Kruskal-Wallis test. Correlation analyses between metric data were carried out using Spearman’s correlation analysis. All results were reported as absolute frequencies, percentages, or means ± standard deviation. A P-value of less than 0.05 was considered statistically significant. All statistical analyses were performed using DATAtab software (DATAtab e.U., Graz, Austria).

## Results

### Patients

A total of 200 patients were included in the study (134 women and 66 men). The mean age was 62.5 ± 12.4 years. Of the participants, 127 individuals tested positive for RF, and 109 were positive for ACPA. At the time of study enrollment, the mean DAS28 score was 3.7 ± 1.4. The following DMARD therapies were utilized: methotrexate (MTX) alone 34.5%; MTX in combination 24.5%; DMARD therapy without MTX 14%; early disease prior to DMARD initiation 23.5%; established disease without DMARD therapy 3.5%. Table 1 summarizes all baseline characteristics of the enrolled individuals.

**Table 1:** Baseline Characteristics of All Included Patients

Variable	Result
Gender (females, males), n (%)	134 (67%), 66 (33%)
Age (± SD), years	62.5 ± 12.4
BMI, kg/m^2^	28.7 ± 5.6
VAS (mean ± SD)	4.0 ± 2.5
HFQ (mean ± SD), %	75.2 ± 22.2
DAS28 (mean ± SD)	3.7 ± 1.4
Swollen small joints (mean ± SD), n	3.1 ± 7.8
Swollen large joints (mean ± SD), n	0.7 ± 1.3
Painful small joints (mean ± SD), n	5.8 ± 9.8
Painful large joints (mean ± SD), n	2.1 ± 2.4
RF, %	64.1
Anti-CCP, %	55.6
Medication	
NSAID therapy, %	28.7
Daily prednisolone (mean ± SD), mg	3.4 ± 2.9
Sulfasalazine, %	12.5
Leflunomide, %	7.5
Anti-TNF-alpha, %	11.0
Anti-IL-6, %	3.0
Anti-CD80/86, %	0
Anti-CD20, %	1.5
CV risk factors/morbidities	
Smoking, %	32.3
Physical exercise, %	42.3
Distress, %	38.9
arterial hypertension, %	65
CAD, %	7
HF, %	4.1
Family history of CAD, %	23
Diabetes mellitus, %	14
Obesity, %	35.5
Laboratory findings	
CRP (mean ± SD), mg/L	6.3 ± 9.8
Hb (mean ± SD), g/L	134.8 ± 22.2
Platelets (mean ± SD), × 100,000/µL	273.6 ± 73.1
Serum creatinine (mean ± SD), µmol/L	71.2 ± 15.9
HbA1c (mean ± SD), %	5.6 ± 0.7
Triglycerides (mean ± SD), mmol/L	1.5 ± 0.9
Total cholesterol (mean ± SD), mmol/L	5.4 ± 1
LDL (mean ± SD), mmol/L	3.2 ± 0.9
HDL (mean ± SD), mmol/L	1.7 ± 0.5

BMI: body mass index; CAD: coronary artery disease; CCP: cyclic citrullinated peptide; CRP: C-reactive protein; CV: cardiovascular; DAS28: Disease Activity Score 28; HDL: high-density lipoprotein; HF: heart failure; HFQ: Hannover Functional Questionnaire; IL-6: interleukin-6; LDL: low-density lipoprotein; NSAID: non-steroidal anti-inflammatory drug; RF: rheumatoid factor; SD: standard deviation; TNF: tumor necrosis factor; VAS: Visual Analogue Scale.

### oxHDL and CVR

No significant correlations or associations were found with the following variables: age (years) (r = −0.06; P = 0.42), gender (P = 0.30), serum creatinine (µmol/L) (r = 0.11; P = 0.13), HbA1c (%) (r = −0.08; P = 0.29), LDL (mmol/L) (r = 0.04; P = 0.55), smoking status (yes/no) (P = 0.38), physical exercise (yes/no) (P = 0.73), distress (yes/no) (P = 0.21), known coronary artery disease (yes/no) (P = 0.74), known heart failure (yes/no) (P = 0.36), family history of atherosclerosis (yes/no) (P = 0.60), and diabetes mellitus (yes/no) (P = 0.18).

**Figure 1:**
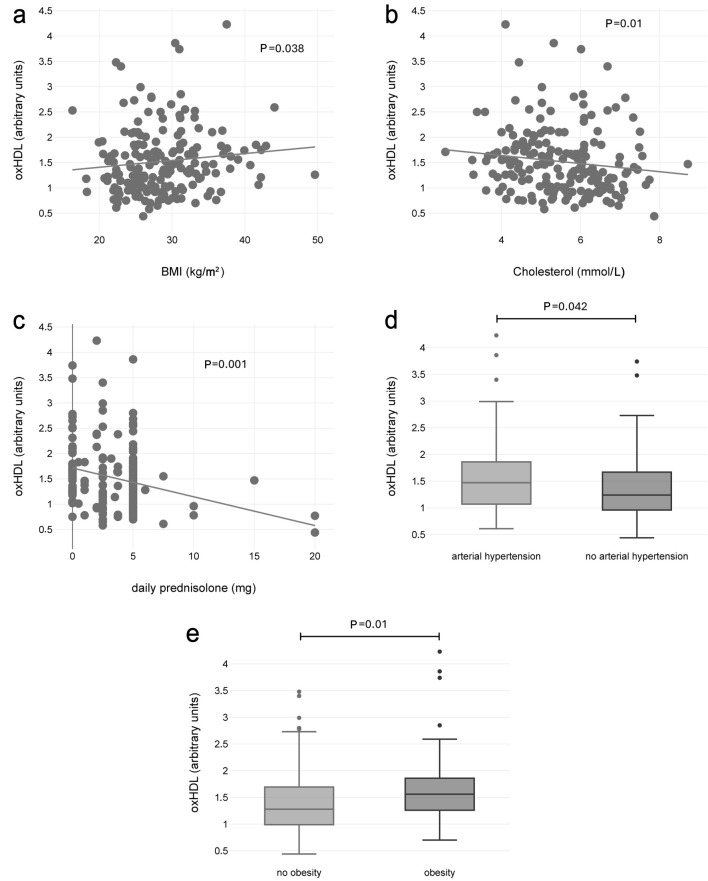
Statistically significant findings from the cardiovascular risk analysis included a positive correlation between oxHDL and BMI (a), as well as negative correlations between oxHDL and total cholesterol (b) and the average daily prednisolone dose (c). Individuals with hypertension (d) and overweight status (e) exhibited higher oxHDL levels. BMI: body mass index; oxHDL: oxidized high-density lipoprotein.

Significant correlations and associations were found with the following variables: BMI (kg/m^2^) (r = 0.15; P = 0.038), average daily prednisone dosage (mg) (r = −0.25; P = 0.001), total cholesterol (mmol/L) (r = −0.17; P = 0.01), arterial hypertension (yes/no) (P = 0.042), and obesity (yes/no) (P = 0.01). Table 2 provides a comprehensive summary of all results, while Figure 1 illustrates the significant findings.

**Table 2:** Cardiovascular Risk Factor Analysis

Variable	Result	P-value
Age (years)	r = −0.06	0.42
Gender (females vs. males)	1.47 ± 0.6 vs. 1.6 ± 0.7	0.3
BMI (kg/m^2^)	r = 0.15	0.038
Average daily prednisone dosage (mg)	r = −0.25	0.001
Serum creatinine (µmol/L)	r = 0.11	0.13
HbA1c (%)	r = −0.08	0.29
Total cholesterol (mmol/L)	r = −0.17	0.01
LDL (mmol/L)	r = 0.04	0.55
Smoking (yes vs. no)	1.4 ± 0.6 vs. 1.5 ± 0.6	0.38
Physical exercise (yes vs. no)	1.5 ± 0.6 vs. 1.5 ± 0.6	0.73
Distress (yes vs. no)	1.6 ± 0.6 vs. 1.4 ± 0.6	0.21
Arterial hypertension (yes vs. no)	1.5 ± 0.6 vs. 1.4 ± 0.6	0.042
Coronary artery disease (yes vs. no)	1.4 ± 0.5 vs. 1.5 ± 0.6	0.74
Heart failure (yes vs. no)	1.5 ± 0.3 vs. 1.4 ± 0.6	0.36
Family history of atherosclerosis (yes vs. no)	1.4 ± 0.5 vs. 1.5 ± 0.6	0.6
Diabetes mellitus (yes vs. no)	1.6 ± 0.7 vs. 1.5 ± 0.6	0.18
Obesity (yes vs. no)	1.6 ± 0.7 vs. 1.4 ± 0.6	0.01

Summary of all correlation and association analyses between oxHDL and the defined nominal and metric variables. oxHDL concentrations are reflected by arbitrary units, respectively. BMI: body mass index; LDL: low-density lipoprotein; oxHDL: oxidized high-density lipoprotein.

### oxHDL and RA disease activity

No significant correlations or associations were found between oxHDL and the following variables: VAS (r = 0.03; P = 0.6), HFQ (%) (r = 0.05; P = 0.47), DAS28 (r = 0.1; P = 0.19), and CRP (mg/L) (r = −0.12; P = 0.1).

**Figure 2:**
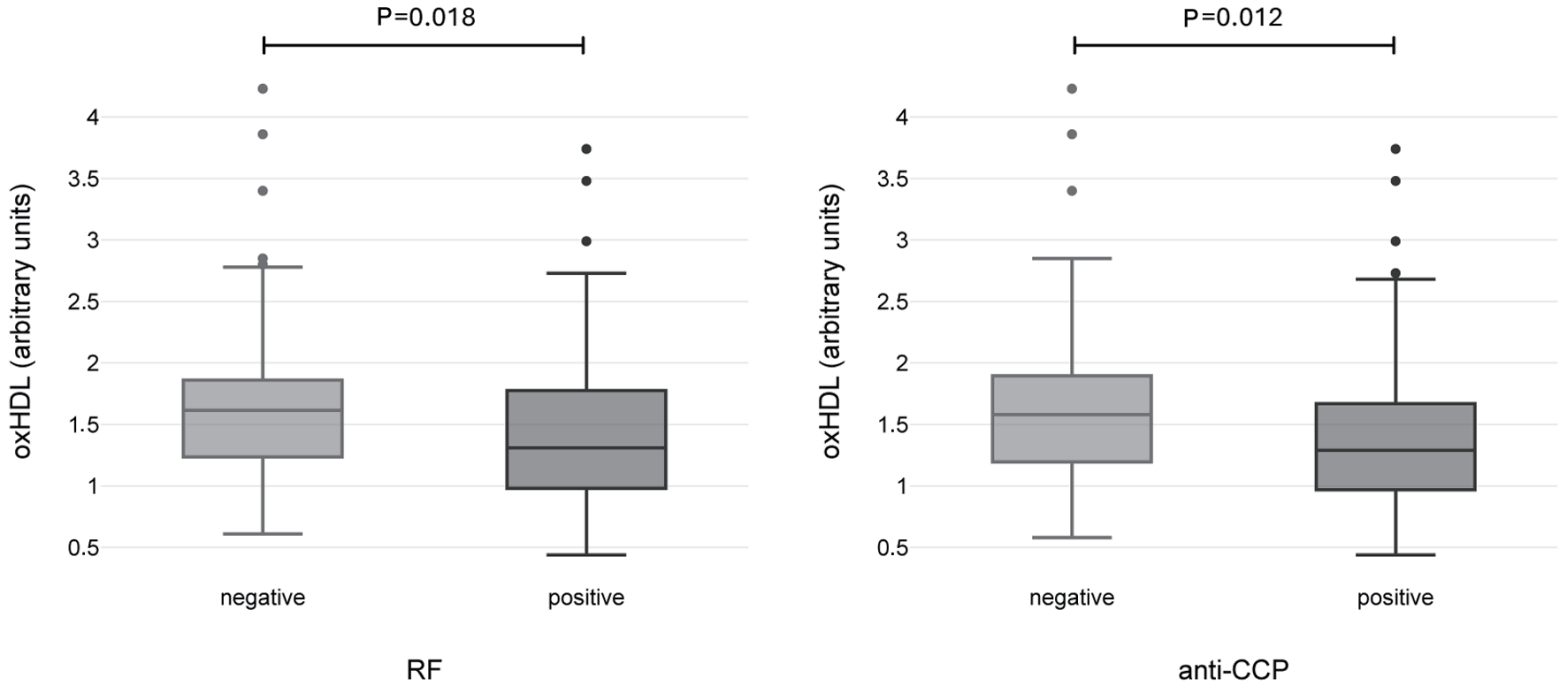
Statistically significant findings from the RA disease activity analysis. Individual with positive RF (a) and positive anti-CCP (b) displayed lower oxHDL, respectively. CCP: cyclic citrullinated peptide; oxHDL: oxidized high-density lipoprotein; RA: rheumatoid arthritis; RF: rheumatoid factor.

Significant correlations/associations were identified between oxHDL and the platelet count (3 × 10^5^/µL) (r = −0.17; P = 0.021). RF-negative individuals exhibited significantly higher levels of oxHDL (P = 0.018), and the same was true for ACPA-negative patients (P = 0.012). In complementary multiple linear regression analyses, the variables platelet count or RF or ACPA were evaluated with the following covariables: BMI, age, gender. oxHDL has been defined as a dependent variable. The following adjusted P-values were obtained: platelet count 0.43, RF 0.046, and ACPA 0.019. Table 3 and Figure 2 summarize the findings.

**Table 3:** RA Disease Activity Analysis

Variable	Result	P-value	Adapted P-value
Visual Analog Scale	r = 0.03	0.6	
Hannover Functional Questionnaire (%)	r = 0.05	0.47	
Disease Activity Score 28	r = 0.1	0.19	
C-reactive protein (mg/L)	r = −0.12	0.1	
Platelet count (× 100,000/µL)	r = −0.17	0.021	0.43
Rheumatoid factor positive (yes vs. no)	1.4 ± 0.6 vs. 1.6 ± 0.7	0.018	0.046
Anti-CCP positive (yes vs. no)	1.4 ± 0.6 vs. 1.6 ± 0.7	0.012	0.019

Summary of all correlation and association analyses between oxHDL and the defined nominal and metric variables. oxHDL concentrations are reflected by arbitrary units, respectively. The last column shows the adapted P-values after multiple linear regression. For this purpose, age, gender, and BMI were chosen as further covariables, oxHDL was the dependent variable. CCP: cyclic citrullinated peptide; oxHDL: oxidized high-density lipoprotein; RA: rheumatoid arthritis.

## Discussion

The primary goal of the investigation was to analyze potential correlations or associations between oxHDL and CVR factors in patients with RA. While relevant findings were undoubtedly identified, the overall pattern of the CVR analysis was inconsistent. Hypertensive and overweight patients exhibited significantly elevated oxHDL levels compared to unaffected individuals, with a positive correlation observed with BMI. Conversely, inverse correlations were found with total cholesterol and the average daily dose of prednisolone. Strictly speaking, an increase in LDL is a well-established CVR factor, yet no positive or negative correlation was observed with this variable. There were no correlations identified with other CVR factors, particularly not with the more potent risk profiles such as diabetes mellitus and chronic kidney disease (as reflected by serum creatinine) [25, 26].

The existing literature presents a rather inconsistent picture regarding the CVR-predictive properties of oxHDL. Nevertheless, there is no doubt that some studies indicated oxHDL as a significant CVR factor. In a prospective investigation involving nearly 900 patients, elevated HDL lipid peroxidation (oxHDL) was associated with confirmed coronary heart disease (CHD). The adjusted odds ratio was 1.69 (95% confidence interval (CI): 1.24–2.38) [27]. In patients undergoing hemodialysis, elevated levels of oxHDL combined with increased interleukin-6 (IL-6) were associated with higher cardiovascular mortality. While oxHDL was not a predictor of overall mortality, it was associated with cardiovascular disease-related events [28]. In a cohort with AF, patients exhibited approximately a 9% higher HDL peroxide level (nHDL_ox). Even after adjustment, this marker remained strongly associated with AF (P ≤ 0.01) [17]. Finally, obese women (BMI > 30) exhibited elevated oxHDL levels, along with inflammatory markers, even in the absence of manifest CHD [17]. However, conflicting findings have also been published. Prospectively, lower oxHDL levels were associated with reduced progression of coronary calcification (Agatston score) [29]. In a cohort of CAD patients, oxHDL demonstrated an inverse association with high-risk plaque features, such as necrotic and fibro-fatty burden. Lower oxHDL levels were linked to unfavorable plaque characteristics. Notably, oxHDL proved to be a better predictor than HDL-C or oxLDL [12]. An explanation for these conflicting results can partially be provided by considering some of the following features: there is no standardized method for measuring oxHDL, as different studies employ various detection techniques (e.g., enzyme-linked immunosorbent assay (ELISA), mass spectrometry) that identify distinct modifications of HDL (such as oxidized ApoA-I, lipid peroxidation of HDL particles, etc.) [30]. Some tests detect mild oxHDL, which may still retain its functionality, while others target heavily modified HDL that has lost its protective properties. OxHDL may still be functional in certain contexts (for example, cholesterol uptake), but when extensively modified, it can exert pro-inflammatory effects [31]. In early stages of atherosclerosis, mild HDL oxidation can serve as an adaptive mechanism, exerting antioxidant or anti-inflammatory effects. However, in later stages or under conditions of chronic oxidative stress, oxHDL can become dysfunctional, developing pro-inflammatory or pro-atherogenic characteristics. However, the final aspect appears to have only some validity, as illustrated by the previously cited study by Sorokin et al [12].

The interpretation of our data is further complicated by the fact that the survey was conducted at a single point in time. Additionally, our study lacks a non-rheumatic control group with comparable characteristics such as age, gender, and other relevant factors. Currently, regarding CVR prediction in RA, it can only be concluded that oxHDL may potentially be helpful under certain conditions in assessing atherosclerosis susceptibility; however, a general suitability as a new CVR factor in RA cannot be established at this time. A final aspect to note concerns the possible effects of glucocorticoids on HDL/oxHDL metabolism. As early as 2020 [32], it was proven that systemic glucocorticoids can result in increased production of dysfunctional HDL. The steroids regularly used in our collective will have potentially had an influence on the results, even if the exact effect is difficult to estimate at the moment.

Regarding the suitability of oxHDL as an additional activity parameter for RA, differences in lipid concentrations were exclusively identified between seropositive and seronegative individuals. No associations were found with established activity indices such as CRP and DAS28. Therefore, it currently cannot be reliably concluded that oxHDL is a dependable candidate for assessing disease activity in RA.

### Limitations

First, the number of participants in the study is limited. It is a cross-sectional snapshot, and therefore, we cannot draw conclusions about cardiovascular morbidity or mortality based on our data. Another limitation is the absence of a non-RA comparison group with similar epidemiological and clinical characteristics.

### Conclusions

OxHDL was associated with several indicators of cardiovascular morbidity. Because the study had a retrospective design, only very limited conclusions can be drawn. The collected data do not support oxHDL as a possible candidate for CVR assessment in RA, both prospective and follow-up data are needed to definitively answer this question.

## Data Availability

The data supporting the findings of this study are available from the corresponding author upon reasonable request.
